# A Successful Outcome of Veno-Venous Extracorporeal Membrane Oxygenation in Obese Patients with Respiratory Failure in the Course of COVID-19: A Report of Two Cases

**DOI:** 10.3390/ijerph19052761

**Published:** 2022-02-27

**Authors:** Jarosław Janc, Lidia Łysenko, Olga Lewandowska, Olimpia Chrzan, Michał Suchański, Marek Gemel, Patrycja Leśnik

**Affiliations:** 1 Department of Anaesthesiology and Intensive Therapy, 4th Military Clinical Hospital, 50-981 Wroclaw, Poland; jjanc@4wsk.pl (J.J.); olga.lewandowska@hotmail.com (O.L.); olimpia2312@wp.pl (O.C.); michal.suchanski@icloud.com (M.S.); 2 Department of Anaesthesiology and Intensive Therapy, Wroclaw Medical University, 50-529 Wroclaw, Poland; lidia.lysenko@umw.edu.pl; 3 Department of Cardiac Surgery, 4th Military Clinical Hospital, 50-981 Wroclaw, Poland; marekgemel@poczta.onet.pl

**Keywords:** COVID-19, SARS-CoV-2, respiratory failure, ECMO, obese patients

## Abstract

The use of extracorporeal membrane oxygenation (ECMO) in patients with respiratory failure in the course of COVID-19 indicates its limited efficacy and high mortality rates. It seems that one of the conditions for the success of veno-venous ECMO (VV ECMO) in obese patients with COVID-19 is the correct qualification and rapid implementation of this method. We present two cases of obese patients with acute respiratory distress syndrome (ARDS) as a result of SARS-CoV-2 infection with the successful use of ECMO. Two 41-year-old obese patients (Case 1: BMI 31.5 kg/m^2^ and Case 2: 44.5 kg/m^2^), with pneumonia and severe respiratory failure in the course of COVID-19, underwent ECMO therapy. The Extracorporeal Life Support Organization (ELSO) guidelines were used to qualify the patients. Due to the persistence of PaO_2_/FiO_2_ rate <80 for 6 h, a decision was made to implement VV ECMO. Both patients were discharged from the intensive care unit (Case 1: on day 35; Case 2: on day 22). Rapid implementation of VV ECMO in middle-aged, obese patients with ARDS in the course of COVID-19 showed a positive outcome.

## 1. Introduction

Coronavirus disease 2019 (COVID-19), caused by severe acute respiratory syndrome coronavirus 2 (SARS-CoV-2), was first diagnosed in China in December 2019. The disease spread very quickly, resulting in the World Health Organization (WHO) declaring the COVID-19 outbreak a global pandemic on 11 March 2020 [1].

Approximately 5% of COVID-19 patients qualify for hospitalization in the intensive care unit (ICU) [2]. The necessity of treating COVID-19 patients in the ICU is associated with a high probability of complications, such as severe respiratory failure, including acute respiratory distress syndrome (ARDS) or patient self-inflicted lung injury (P-SILI) [3,4,5]. In a small proportion of patients with severe respiratory failure, optimal mechanical ventilation does not provide adequate gas exchange and can lead to ventilator-induced lung injury (VILI) [6]. Despite the implementation of the recommended procedure, patients continue to experience persistent hypoxemia, in some cases with concomitant hypercapnia. Veno-venous extracorporeal membrane oxygenation (VV ECMO) then becomes the only life-saving treatment option [7,8].

Based on the studies published so far, ECMO therapy in severe, resistant to conventional treatment SARS-CoV-2 infection is a life-saving treatment option, considering contraindications such as advanced age, multi-organ failure, damage to the central nervous system, and mechanical ventilation time longer than 10 days [8]. Obesity is one of the risk factors for a severe course of COVID-19. About 50% of patients on the Extracorporeal Life Support Organization (ELSO) registry are obese, with a body mass index (BMI) > 30 kg/m^2^. Class III obesity, which is defined as a BMI of more than 40 kg/m^2^ according to the WHO, is included in the ELSO COVID-19 Interim Guidelines of relative contradictions to ECMO [2,8,9,10]. The difficulties in cannulation and obtaining adequate blood flow may be a challenge in these patients.

This study aimed to describe the successful use of ECMO in two obese patients with severe ARDS as a result of SARS-CoV-2 infection.

## 2. Case Report 1

**Figure 1:**
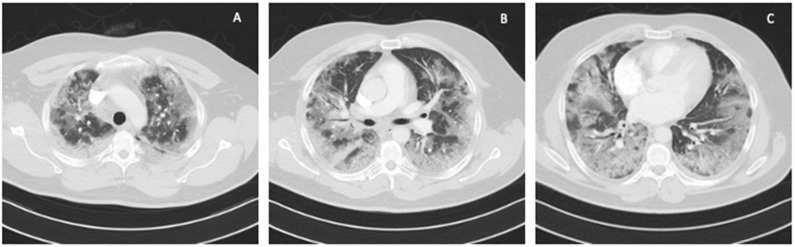
Chest CT scan on admission to the ICU: (**A**) upper lung segments; (**B**) middle lung segments; (**C**) basal lung segments (Case 1).

A 41-year-old obese man with BMI = 31.5 kg/m^2^ (class I obesity), without comorbidities, was admitted to the ICU due to symptoms of respiratory failure worsening as a result of SARS-CoV-2 infection. On admission to the ICU, a chest CT angiography scan was performed, showing signs of pulmonary embolism and ground-glass opacity inflammatory lesions, affecting up to 80% of the lung parenchyma (Figure 1).

In the ICU, intravenous infusion of dexmedetomidine and therapeutic doses of low-molecular-weight heparin were used; the patient was not qualified for treatment with remdesivir (disease duration longer than 7 days) in accordance with the National Institutes of Health (NIH) guidelines for COVID-19 treatment [11,12,13]. Respiratory support was performed using non-invasive ventilation (NIV) through an oronasal mask in PS/CPAP mode with a fraction of inspired oxygen (FiO_2_) 1.0, obtaining PaO_2_/FiO_2_ ratio <65. Two days after admission to the ICU, due to a decrease in blood saturation (SaO_2_) <85% and in oxygen pressure in arterial blood (PaO_2_) to 54 mmHg, severe dyspnea and tachypnea up to 40 BPM, the patient was intubated, invasive mechanical ventilation in the SIMV (PC) mode with FiO_2_ of 1.0, PEEP 10 cmH_2_O was initiated, achieving optimal minute ventilation (MV), which is defined as tidal volume (V_T_) calculated for ideal body weight (IBW) x respiratory rate (f) [14]. Dynamic compliance (C_dyn_) was 40 mL/cmH_2_O, inspiratory pressure (P_ins_) was 23 cmH_2_O, and plateau pressure (P_plat_) was 20 cmH_2_O. Multimodal deep analgosedation was included. Recruitment maneuvers were used, consisting of inspiration extended to 20 s with P_plat_ up to 30 cmH_2_O, performed twice. The PaO_2_ value decreased to 66.2 mmHg, and the pressure of carbon dioxide in arterial blood (PaCO_2_) increased to 68 mmHg. Considering the serious technical difficulties related to the significant abdominal obesity of the patient, no attempts were made to put the patient in the prone position. Since the ELSO criterion for PaO_2_/FiO_2_ ratio <80 for 6 h was met, the patient was qualified for VV ECMO on day 2 of treatment in the ICU. The calculated Respiratory ECMO Survival Prediction (RESP) score [15] was 6, with a class II risk and an estimated ECMO survival probability of 92%.

**Figure 2:**
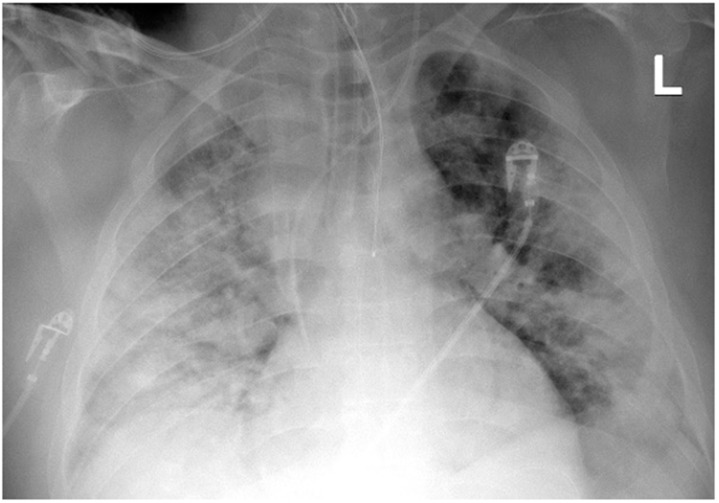
Chest X-ray after insertion of the 21F cannula into the superior vena cava (Case 1).

The Seldinger technique was used to place a 15-cm long, 21F-thick BIOLINE-coated Getinge HLS cannula into the superior vena cava through the right internal jugular vein (inflow), and a 65-cm long, 25F-thick BIOLINE-coated Getinge HLS cannula was inserted surgically through the right femoral vein into the inferior vena cava (intake). The surgical technique was applied because of the patient’s significant obesity and the expected technical difficulties in administering femoral vein venipuncture. The correct position of the cannulas was confirmed by ultrasound examination and chest X-ray (Figure 2).

A Medtronic Bio-Console 560 with a Medtronic Bio-Pump 540T and a Eurosets EU5520 oxygenator were used. Extracorporeal oxygenation was initiated, with a pump minute output of 6.5 L/min, RPM of 4540, and a 100% O_2_ sweep gas flow of 8 L/min_._ The procedure was performed using anticoagulation therapy with unfractionated heparin by intravenous infusion under the control of activated clotting time (ACT) in the range of 200–230 s. After ECMO was initiated, lung-protective mechanical ventilation was conducted in the SIMV (PC)+ VS mode, PEEP 10–15 cmH_2_O, Pplat ≤ 25 cmH_2_O, respiratory rate (f)-6/min, driving pressure (∆P) < 15 cmH_2_O, FiO_2_ < 0.5, and tidal volume (VT) ≤ 6mL/kg predicted body weight (PBW). PaO_2_ was 72 mmHg and PaCO_2_ 37 mmHg. Initially, it was not possible to obtain satisfactory values of capillary blood saturation, despite the use of optimal pump operation parameters. The position of the cannulas was checked with an ultrasound examination. The intake cannula was found to be too high, and it was withdrawn, positioning it approximately 5 cm below the entrance of the inferior vena cava to the right atrium. Due to the abnormal values of the pressures in the ECMO system, gradual anemization of the patient, and the appearance of hemolysis markers (increased levels of bilirubin and myoglobin in the blood), oxygenator clotting was suspected. For this reason, the ECMO system was replaced, resulting in an improvement in oxygenation and withdrawal of the above-mentioned symptoms. During the ECMO therapy, the patient periodically required stabilization of the arterial pressure with an intravenous infusion of noradrenaline. The initial measure of C_dyn_ was 25 mL/cm H_2_O with a low VT of about 250 mL. These parameters gradually increased: C_dyn_ to 40 mL/cm H_2_O and VT to 450 mL, respectively. In the initial period of ECMO use, lung ultrasonography (LUS) was characterized by a bilateral, confluent occurrence of the B-line, a thickened and indented pleural line, and massive basal consolidations. From day 9 of treatment, the LUS image began to improve, and A-lines gradually appeared. The need for prolonged mechanical ventilation for 7 days resulted in the patient undergoing percutaneous Griggs tracheostomy. Ceftazidime-avibactam (Zavicefta) was added to the treatment for 7 days due to the increase in the parameters of inflammation and symptoms of infection, as well as the diagnosis of ventilator-associated pneumonia (VAP) with ESBL-producing *Klebsiella pneumoniae* etiology. Consequently, a significant decrease in inflammatory parameters such as procalcitonin (PCT) and C-reactive protein (CRP) as well as negative microbiological test results was achieved.

On day 13 of ECMO use, the pump minute output was decreased to 3 L/min and the 100% O_2_ sweep gas flow was gradually reduced by 2 L/min every 30 min. The supply of all gases to the oxygenator was completely stopped, and the patient was mechanically ventilated with FiO_2_ of 0.5. The patient’s condition was monitored for another 60 min, after which time PaO_2_ of 110 mmHg and PaCO_2_ of 49 mmHg were obtained. The flow of the ECMO pump was consequently stopped, and both cannulas were removed. In the following days, lung-protective mechanical ventilation was continued in the SIMV (PS) mode. After 9 consecutive days, the PS/CPAP mode with FiO_2_ of 0.35 was used, and intensive rehabilitation began. The patient experienced withdrawal symptoms after discontinuation of opioids: intention tremors, profuse sweating, and tachypnea. Due to the hypotension tendency, no clonidine derivatives were included in the treatment; methadone was administered, achieving resolution of the withdrawal symptoms. The patient also experienced increased anxiety, difficulty falling asleep, and nightmares. After a psychiatric consultation, sertraline was introduced, and the patient had regular therapeutic sessions with a psychologist. Significant improvements in mood and withdrawal of anxiety symptoms were achieved. On day 16 of treatment in the ICU, RT-PCR testing for SARS-CoV-2 was performed; the result was negative. The tracheostomy tube was removed after 29 days of the treatment in the ICU (15 days after ECMO discontinuation). Finally, on day 35 of treatment in the ICU (21 days after ECMO discontinuation), the patient, assessed at 80 points in the Barthel Index [16], was discharged from the ICU and transferred to a rehabilitation center for further treatment.

## 3. Case Report 2

**Figure 3:**
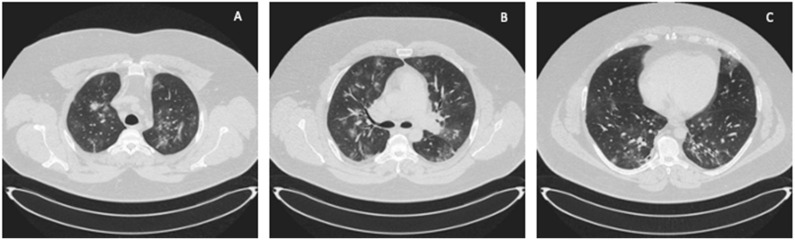
ICU: (**A**) upper lung segments; (**B**) middle lung segments; (**C**) basal lung segments (Case 2).

A 41-year-old obese man with a BMI of 44.5 kg/m^2^ (class III obesity) was admitted to the ICU on day 2 after admission to the hospital due to respiratory failure in the course of SARS-CoV-2 infection. The patient had comorbidities: type 2 diabetes mellitus and obstructive sleep apnea. On admission to the hospital, a CT scan of the lungs showed lesions typical of COVID-19 (Figure 3).

**Figure 4:**
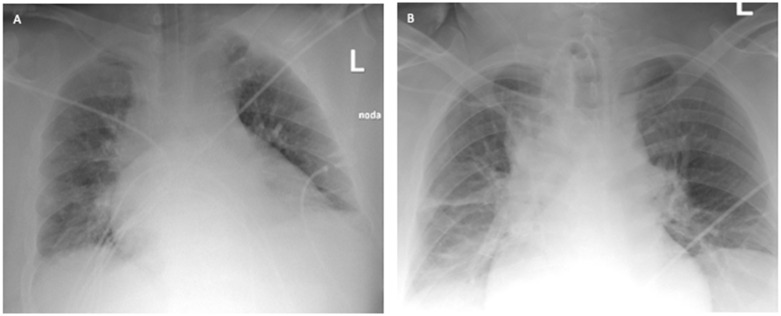
Chest X-ray: (**A**) after admission to the ICU; (**B**) 2 days after starting VV ECMO treatment (Case 2).

The ICU team used NIV through an oronasal mask in PS/CPAP mode with FiO_2_ of 1.0. Due to the increasing symptoms of respiratory failure (PaO_2_ = 61 mmHg, PaCO_2_ = 66.6 mmHg), dyspnea, and tachypnoea >40 BPM, as well as significant respiratory effort and disturbances of consciousness, the patient was intubated, and 4 h after admission to the ICU, mechanical ventilation was initiated in the SIMV (PC) mode with FiO_2_ of 0.9, PEEP = 13 cmH_2_O, P_ins_ = 24 cmH_2_O, C_dyn_ = 46 mL/cmH_2_O, P_plat_ = 21 cmH_2_O, obtaining optimal MV [14]. Deep hypoxemia with a PaO_2_/FiO_2_ ratio <60 was observed. An infectious disease specialist did not qualify the patient for treatment with remdesivir. Multimodal deep analgosedation was included. Dexamethasone IV at a dose of 6 mg, as per NIH guidelines, and therapeutic doses of low-molecular-weight heparin were used [12]. LUS imaging showed a bilateral B-profile and massive basal consolidations. The cardiac ultrasound demonstrated the proper function of the right and left ventricles, and no valvular changes were recorded. On day 2 from the initiation of the mechanical ventilation, the prone position maneuver was attempted; however, it was unsuccessful due to the difficulties related to the patient’s abdominal obesity. Additionally, numerous recruitment maneuvers were used, consisting of inspiration extended to 20 s with P_plat_ up to 30 cmH_2_O, performed twice, without improvement in oxygenation or increase in C_dyn_. On day 4 of the ICU stay, due to PaO_2_/FiO_2_ ratio <60, a decision was made to use VV ECMO. The calculated RESP score was 4, with a class II risk and an estimated ECMO survival probability of 76%. Before cannulation, the patient underwent percutaneous Griggs tracheostomy. The implantation technique and cannulation equipment used were identical to those described in Case 1. The correct position of the cannulas was confirmed by ultrasound examination and chest X-ray (Figure 4).

Using identical Medtronic equipment, extracorporeal oxygenation was initiated, with a pump minute output of 5.3 L/min, RPM 4520/min, and a 100% O_2_ sweep gas flow of 8 L/min. The procedure was performed with anticoagulation therapy with unfractionated heparin by intravenous infusion under the control of ACT in the range of 200–230 s. Initial ECMO settings were adjusted according to the following lung-protective ventilator strategy: SIMV (PC), VT < 6 mL/kg PBW, P_plat_ < 25 cmH_2_O, PEEP 10 cmH_2_O, f = 10/min, ∆P < 15 cmH_2_O, and FiO_2_ < 0.5. The patient was hemodynamically stable and did not require the administration of catecholamines. From day 3 of the VV ECMO therapy, the patient’s condition gradually improved. The 100% O_2_ sweep gas was reduced, there was a significant increase in C_dyn_ to 70 mL/cmH_2_O and PaCO_2_ was 40 mmHg. On day 7 from the start of VV ECMO treatment, the RPM was decreased to 2600/min, gradually reducing the 100% O_2_ sweep gas by 2 L/min every 30 min. In the upper and lower fields, LUS imaging showed a bilateral A-profile without consolidations. On day 11 of the ICU stay, VV ECMO was discontinued, and both cannulas were removed. The procedure of bringing the patient out of sedation was commenced, initially using mechanical ventilation in the SIMV (PC) mode with FiO_2_ of 0.4, PEEP = 12 cmH_2_O, C_dyn_ > 100 cmH_2_O and dexmedetomidine infusion. No delirium symptoms were observed, and after 24 h, the dexmedetomidine infusion was terminated. On day 13 of the ICU stay (2 days after ECMO discontinuation), after full awakening of the patient, the mode of ventilation was changed to PS/CPAP 10 cmH_2_O, with FiO_2_ of 0.35. On day 14 of the treatment (3 days after ECMO discontinuation), the patient had the tracheostomy tube removed. Finally, on day 22 of treatment in the ICU (11 days after ECMO discontinuation), the patient, assessed at 70 points in the Barthel Index, was discharged from the ICU and transferred to a rehabilitation center for further treatment.

**Figure 5:**
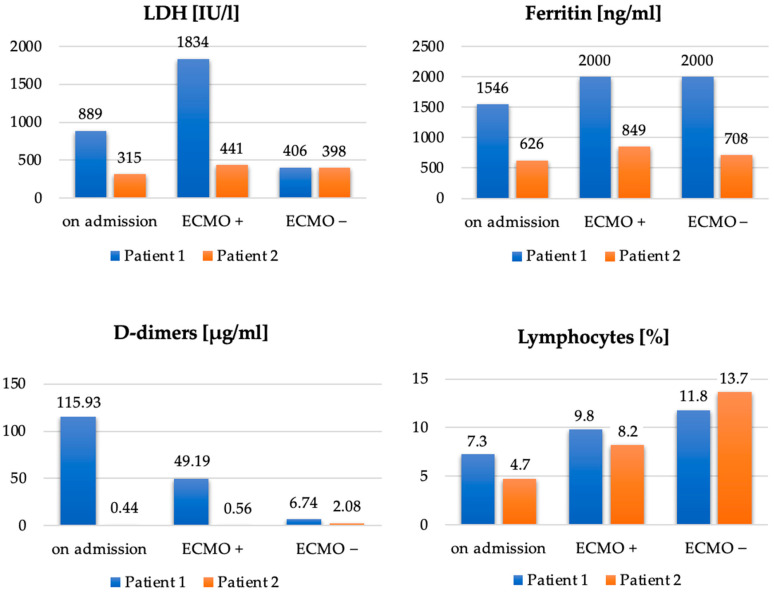
Comparison of LDH [IU/L], ferritin [ng/mL], D-dimers [µg/mL] and lymphocytes [%] in Patient 1 and Patient 2, on admission to the ICU, after initiation of ECMO (ECMO+) and on discontinuation of ECMO (ECMO–).

In summary, Table 1 and Figure 5 show a comparison of the main points of the clinical course and the selected laboratory parameters of both described cases.

## 4. Discussion

This paper presents the successful use of ECMO in two patients with obesity and treatment-resistant respiratory failure in the course of COVID-19. Obese patients suffer from numerous disorders in the respiratory system. Furthermore, a high BMI is often associated with insulin resistance, type 2 diabetes mellitus, arterial hypertension, coronary artery disease, obstructive sleep apnea syndrome, and hypoventilation. Such numerous BMI-related issues may present a negative prognosis for obese patients undergoing VV ECMO treatment. However, there are studies in which this dependence has not been demonstrated [17,18]. In the case of ARDS occurring in the course of SARS-CoV-2 infection, both the WHO and ELSO recommend the use of ECMO in treatment-resistant hypoxemia despite the use of lung-protective mechanical ventilation [19]. As mentioned earlier, class III obesity is included in the ELSO COVID-19 Interim Guidelines of relative contradictions to ECMO. In many health centers, the use of ECMO in the group of obese patients raises doubts. The difficulties in cannulation and obtaining adequate blood flow may be a challenge in these patients. Ramirez et al. [20] showed that ECMO therapy provided to 33 COVID-19 patients with BMI > 40 benefited from ECMO support and had similar outcomes and a similar mortality rate to patients who were not morbidity obese. Furthermore, Carlese et al. [21] showed a very good outcome of VV-ECMO treatment in 13 extremely obese patients. However, the fact is that more extensive studies are needed to exclude elevated BMI as a relative contraindication for ECMO. Moreover, Shere et al. [22] demonstrated a case study of a 54-year-old male reporting a significant advantage of VV ECMO using direct pulmonary artery flow, which improved oxygenation and ventilation with a survival benefit.

In the described cases of two 41-year-old obese men with class I and class III obesity pneumonia and severe respiratory failure in the course of COVID-19, we demonstrated the effectiveness of ECMO therapy. Imaging studies of both patients showed that over 70% of the bilateral lung parenchyma was affected by COVID-19 lesions. Due to the patients’ obesity, using the prone position was technically difficult and associated with a high risk of complications. Interestingly, a more favorable course of the disease was observed in Case 2, that is, in a patient with a higher class of obesity (Table 1, Figure 5). Compared to Case 1, the use of ECMO was shorter, the patient was disconnected from the ventilator and decannulated faster, and his ICU stay was shorter. This patient showed lower impairment of significant laboratory parameters, such as LDH, ferritin, and D-dimers. Upon discontinuation of ECMO, a lower degree of lymphopenia was also observed in Case 2 compared to Case 1. The longer ECMO therapy in Case 1 was probably due to bacterial superinfection of the lungs caused by an ESBL *K*. *pneumoniae* strain; rapid implementation of effective antibiotic therapy resulted in an improvement in ventilation parameters and a decrease in inflammation indexes. A patient with a BMI > 40 and more accompanying diseases (Case 2) was treated for a shorter time because it was possible to avoid complications in the form of bacterial pneumonia. Therefore, careful diagnostics and rapid implementation of adequate antibacterial treatment should be the standard of care during ECMO therapy.

In both patients, therapy was implemented within the first 5 days of the ICU treatment, and <96 h after intubation and the initiation of mechanical ventilation. Early inclusion of ECMO may have reduced the potential risk of patient self-induced lung injury (P-SILI) observed in patients breathing with significant respiratory effort [4,5]. Despite the use of high positive end-expiratory pressure (PEEP) and recruitment maneuvers, no improvement was achieved during mechanical ventilation. Because of the persistence of PaO_2_/FiO_2_ ratio <80 for 6 h, a decision was made to implement VV ECMO. The femoral cannula was surgically inserted in both patients due to the expected technical difficulties. In Patient 1, delirium symptoms were observed after discontinuation of multimodal analgosedation and resolved rapidly with methadone therapy. The second patient showed no withdrawal symptoms.

In both described patients, the use of ECMO was the last resort therapy. After discontinuation of ECMO, the patients were weaned from the ventilator with the use of percutaneous tracheostomy performed earlier and with intensive rehabilitation. On discharge from the ICU, both patients achieved between 70 and 80 points in the Barthel Index, which proves that the therapy was successful. In our two cases, ventilation during ECMO with decreased Pins and driving pressure reduced the potential risk of VILI [6].

## 5. Conclusions

The presented description of two patients with obesity and severe respiratory failure in the course of COVID-19 shows the positive effect of the quick implementation of VV ECMO. These successfully treated cases confirm the thesis that the group of patients with obesity should not be deprived of the option of using ECMO. There is certainly a need for further evaluation and research into the use of ECMO in non-standard groups of patients with COVID-19, including patients with obesity.

## Figures and Tables

**Table 1:** The main points of the clinical course of the described cases.

	Day after Admission to ICU	
	Case 1	Case 2
Intubation	2	1
V-V ECMO initiation	2	4
V-V ECMO discontinued	13	11
Decannulation	29	14
Discharged from ICU	35	22

## Data Availability

The data presented in this study are available on request from the corresponding author.
